# Regulation of ZMYND8 to Treat Cancer

**DOI:** 10.3390/molecules26041083

**Published:** 2021-02-18

**Authors:** Yun Chen, Ya-Hui Tsai, Sheng-Hong Tseng

**Affiliations:** 1 Department of Surgery, Far Eastern Memorial Hospital, Pan-Chiao, New Taipei 220, Taiwan; ychen@mail.femh.org.tw; 2 Department of Chemical Engineering and Materials Science, Yuan Ze University, Chung-Li, Taoyuan 320, Taiwan; 3 Department of Surgery, National Taiwan University Hospital, Taipei 100, Taiwan

**Keywords:** ZMYND8, tumorigenesis, epigenetic regulation, pro-oncogenic effects, tumor suppression

## Abstract

Zinc finger myeloid, nervy, and deformed epidermal autoregulatory factor 1-type containing 8 (Zinc finger MYND-type containing 8, ZMYND8) is a transcription factor, a histone H3-interacting protein, and a putative chromatin reader/effector that plays an essential role in regulating transcription during normal cellular growth. Mutations and altered expression of ZMYND8 are associated with the development and progression of cancer. Increased expression of ZMYND8 is linked to breast, prostate, colorectal, and cervical cancers. It exerts pro-oncogenic effects in breast and prostate cancers, and it promotes angiogenesis in zebrafish, as well as in breast and prostate cancers. In contrast, downregulation of ZMYND8 is also reported in breast, prostate, and nasopharyngeal cancers. ZMYND8 acts as a tumor suppressor in breast and prostate cancers, and it inhibits tumor growth by promoting differentiation; inhibiting proliferation, cell-cycle progression, invasiveness, and metastasis; and maintaining the epithelial phenotype in various types of cancers. These data together suggest that ZMYND8 is important in tumorigenesis; however, the existing data are contradictory. More studies are necessary to clarify the exact role of ZMYND8 in tumorigenesis. In the future, regulation of expression/activity of ZMYND8 and/or its binding partners may become useful in treating cancer.

## 1. Characteristics and Functions of ZMYND8

**Figure 1:**
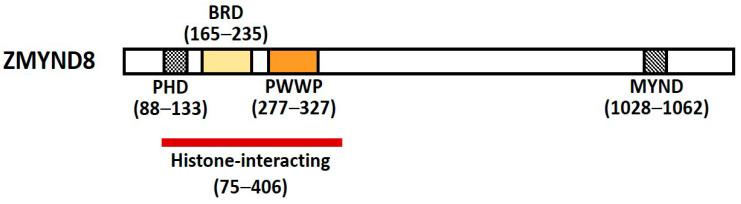
Schematic representation of ZMYND8. ZMYND8 domains include the N-terminal PHD/BRD/PWWP reader cassette and a C-terminal MYND interacting domain. MYND: Myeloid, nervy, and deformed epidermal autoregulatory factor 1; ZMYND8: Zinc finger MYND-type containing 8; PHD: Plant homeodomain; BRD: Bromodomain; PWWP: Pro-Trp-Trp-Pro.

Zinc finger myeloid, nervy, and deformed epidermal autoregulatory factor 1-type containing 8 (Zinc finger MYND-type containing 8, ZMYND8) is a multifunctional transcription factor harboring conserved chromatin-binding module with affinity for chromatin [1]. It was initially identified as activated protein-kinase-C (PKC)-binding protein and is a member of the receptor for activated C-kinase (RACK) family proteins that anchor activated PKC and increase its phosphorylation and duration of inactivation; it is also called RACK7 [2,3]. ZMYND8 contains a Pro-Trp-Trp-Pro (PWWP) chromatin-binding domain, a bromodomain (BRD), a plant homeodomain (PHD) type zinc finger, and a MYND domain for protein–protein interaction (Figure 1) [3,4]. The PHD–BRD–PWWP (PBP) domains are histone readers, and the proteins containing PBP domains have various chromatin-related functions [5].

ZMYND8 is a core chromatin reader/effector, with distinct affinity for histone H3 and H4 [2,4,5,6]. The N-terminal PHD–BRD–PWWP domains of ZMYND8 can read several acetyl and methyl lysine residues on histones, including acetyl lysine 14 of H3 (H3K14ac), H4K16ac, and di- and tri-methyl lysine 36 of H3 (H3K36me2, H3K36me3) [6,7]. The PHD–BRD–PWWP domains of ZMYND8 form a stable structural reader ensemble and simultaneously engage histones and DNA, and then ZMYND8 is recruited to the transcriptional sites of the chromatin [7]. Mutation of the reader ensemble may affect the binding of ZMYND8 to histones by disrupting the interaction interface or destabilizing the domain topology [7]. ZMYND8 has been found to regulate gene expression by recognizing dual histone marks [8]. It regulates the expression of all-trans retinoic acid (ATRA)-responsive genes through specific recognition of H3K36me2/H4K16ac [9,10]. It also recognizes H3K4me1/H3K14ac in DU154 and CWR22Rv1 prostate cancer cells [8], as well as H3K36me2/H4K16ac in SH-SY5Y neuroblastoma cells [4,9]. The dual recognition of two different histone modification by ZMYND8 suggests that the two separate conserved domains PWWP and BRD have different affinities towards their cognate histone binding partners [11]. When both H3K36me2 and H4K16ac exist in same histone octamer, the initial binding and recognition of the ZMYND8 to chromatin is considered through H3K36me2 because of its higher association rate, and the stability of the ZMYND8–nucleosome complex relies more on the binding to H4K16ac, due to its lower association rate [11].

**Figure 2:**
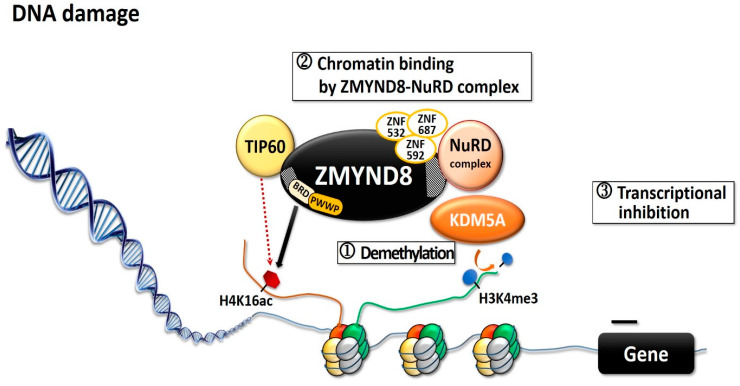
Diagram showing the KDM5A–ZMYND8–NuRD pathway during DNA damage. Upon DNA damage, the hisTable 5. A. ZMYND8 interacts with the NuRD complex and recognizes the TIP60-dependent acetylation. Then ZMYND8–NuRD complex binds to the DNA damaged site, inhibits transcription, and promotes DNA repair. TIP60: Histone acetyltransferase Tat-interactive protein-60KDa; NuRD: Nucleosome remodeling and deacetylase; KDM5A: Lysine demethylase 5A; H4K16ac: Acetyl lysine 16 of H4; H3K4me3: Tri-methyl lysine 4 of H3.

ZMYND8 is involved in transcription activation and in regulating transcription initiation through its interaction with the RNA polymerase II complex [4,12]. Through its putative coiled-coil domain, ZMYND8 forms a homodimer that preferentially associates with positive transcription elongation factor b (P-TEFb) complex, whereas the monomer associates with the chromodomain helicase DNA-binding protein 4 (CHD4) subunit of repressor nucleosome remodeling and deacetylase (NuRD) complex [4,12,13]. ZMYND8 and NuRD share a large number of genome-wide binding sites, mostly in active promoters and enhancers [14]. Both ZMYND8 and CHD4 modulate the expression of many genes and maintain genome integrity; silencing any one of them can alter global gene expression [4,12]. Silencing of ZMYND8 in HeLa cells increases the expression of 331 genes and decreases that of 438 genes [4]. ZMYND8 is important in modulating chromatin integrity and DNA repair [6,15,16]. Upon DNA double-strand break (DSB), histone modifications are altered to accommodate the DNA-damage signaling and the repair [6,15], and BRD2 protein and ZMYND8 are recruited to the DNA damages sites [17]. BRD2 occupies a spatially restricted region extending 2 kb either side of the DSB, and ZMYND8 spreads along the flanking chromatin [17]. The hyperacetylated chromatin domain is required for DBS repair, and the binding of BRD2 to H4ac protects the underlying acetylated chromatin from attack by histone deacetylases, whereas ZMYND8 is a repressor factor which limits transcription during DSB repair [17]. This creates a spatially restricted H4ac/BRD domain which facilitates DSB repair [17]. ZMYND8 interacts with various chromatin-remodeling complexes, histone demethylases/deacetylases, and acetyl transferases, including lysine demethylase 1A (KDM1A), KDM5A, KDM5C, and KDM5D, as well as histone acetyltransferase Tat-interactive protein-60KDa (TIP60) [4,6,8,12,16,18]. KDM5A-dependent demethylation is crucial for the binding of the ZMYND8–NuRD complex to chromatin and its recruitment to the locations of DNA damage (Figure 2) [15]. KDM5A causes H3K4me3 demethylation within chromatin, near the sites of DSB, while ZMYND8, NuRD complex, and KDM5A interact to repress transcription upon DNA damage [6,15]. KDM5A deficiency impairs the transcriptional silencing and the repair of DSBs by homologous recombination [15]. ZMYND8 also interacts with the NuRD complex and TIP60, to mediate DNA repair through homologous recombination [6,16]. In Xenopus embryos, ZMYND8 interacts with RE1-silencing transcription factor corepressor 2 (RCOR2), and together they function as transcriptional repressors in regulating neural differentiation [19]. Both ZMYND8 and p53 play a role in DSB repair [6,15,16]. In several breast cancer cells with distinct p53 genotypes, ZMYND8 loss induced consistent micronucleus formation and DNA-damage response [20]. Additionally, in ZMYND8-depleted human U2OS osteosarcoma cells, laser micro-irradiation induced sustained p53 phosphorylation, which is a DSB marker; in contrast, there was no sustained p53 phosphorylation in control cells [6]. These results imply ZMYND8 and p53 may function independently for repair of DNA damage.

As ZMYND8 is important for transcriptional regulation and chromatin integrity, it may have a role in oncogenesis. However, the reports regarding the influence of ZMYND8 on cancers are ambiguous [2,8,9,21,22,23,24,25]. In this review, we summarize the evidence for both pro-oncogenic and tumor-suppressive effects of ZMYND8 in various types of cancer. We conducted a PubMed literature search, using a combination of the following keywords and their variants: ZMYND8, RACK7, cancers, neoplasms, oncogenesis, carcinogenesis, and epigenetic regulation (up to November 30, 2020). The search covered all English articles listed in PubMed. The titles and abstracts of the identified articles concerning ZMYND8, RACK7, cancer, angiogenesis, proliferation, invasiveness, metastasis, and tumor growth were included. The selected articles were read in full, and further articles that were identified from their reference lists were also reviewed, to include studies that may have been missed in the initial search. A total of 39 references were thus included in the present review.

## 2. Association of ZMYND8 and Cancers

Many epigenetic effectors, including ZMYND8, contain structurally conserved domains of PHD fingers, and alterations in the PHD finger-containing proteins are linked to cancer [4,6,26,27]. ZMYND8 is essential for regulating transcription during normal cellular growth and DNA repair, the perturbation of which may promote cancer initiation and progression [6,15,16]. In fact, ZMYND8 is a cutaneous T-cell lymphoma-associated antigen [28]. In addition, the ZMYND8-v-rel reticuloendotheliosis viral oncogene homolog A (avian) (RELA) chimeric transcripts were reported in a four-month-old patient with acute erythroid leukemia, a type of acute myeloid leukemia (AML) [29]. This fusion gene was thought to be a possible cause of constitutive activation of nuclear factor-kB in AML cells, since the RELA gene was under the control of the ZMYND8 promoter [29]. In breast cancer cells, ZMYND8 may be present as a fusion protein with centrosomal protein 250 (CEP250), which is required for centriole–centriole cohesion during interphase [3,30]. However, the ZMYND8-CEP250 fusion mRNA was not reported in the 111 breast cancer specimens studied [3]. Mutation frequency of *ZMYND8* was 19% in mismatch repair-deficient colorectal cancers [31]. In high-grade serous ovarian cancer, the *ZMYND8* gene is located within a region with recurrent alterations of somatic copy number [32]. An increased copy number (two to six copies) of *ZMYND8* is also found in DU-145, PC-3, LNCaP-FGC, BPH-1, and 22RV1 prostate cancer cells [2]. All of these findings indicate that ZMYND8 may be involved in development and progression of cancer.

## 3. ZMYND8 and Histone Modification in Cancer Cells

**Figure 3:**
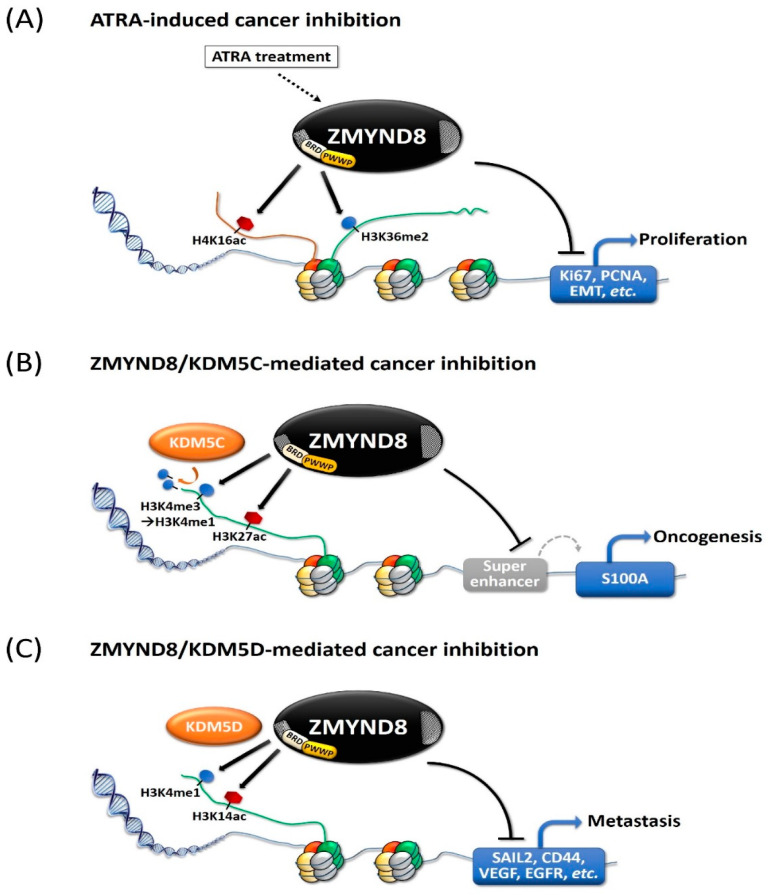
Diagrams showing the mechanisms of the tumor-suppression effects of ZMYND8 and demethylases. The tumor-suppression effects may involve recognition of dual histone marks. (**A**) The all-trans retinoic acid (ATRA)-induced inhibition of transcription of proliferation genes and epithelial–mesenchymal transition (EMT). (**B**) ZMYND8 cooperates with KDM5C to suppress super-enhancer and transcription of oncogenes in breast cancer. (**C**) ZMYND8 cooperates with KDM5D to suppress transcription of metastasis genes in prostate cancer. PCNA: Proliferating cell nuclear antigen; VEGF: Vascular endothelial growth factor; EGFR: Epidermal growth factor receptor.

The function of ZMYND8 in cancer cells is mainly through modulation of histone methylation and acetylation [4,9,11,25,32]. It selectively recognizes H3K36me2/H4K16ac and regulates all-trans retinoic acid (ATRA)-responsive genes in SH-SY5Y neuroblastoma cells [4]. In MDA-MB-231 breast cancer cells, ZMYND8 is recruited to its target genes by binding to H3K36me2 and H4K16Ac (Figure 3A) [11]. Furthermore, in HeLa and MCF7 breast cancer cells, ATRA induces an H3K27me3 to H3K27ac switch and upregulates ZMYND8 expression [9]. Modulation of histone methylation and acetylation in the enhancer regions by ZMYND8 is particularly important, as the dysregulation of this process may cause over-activation of transcription and contribute to tumorigenesis [25]. ZMYND8 and the KDM5 family cooperatively act on super-enhancer regions and are crucial regulators of expression and repression of oncogenes and tumor-suppressor genes in various types of cancer [8,12,16,18,25,33,34,35]. In ZR-75-30 breast cancer cells, ZMYND8 promotes the recruitment of KDM5C to the super-enhancer region, shown by the co-binding of ZMYND8 and KDM5C to 88.7% super-enhancers (Figure 3B) [25]. Ablation of either ZMYND8 or KDM5C in ZR-75-30 breast cancer cells results in over-activation of their target enhancers, characterized by the deposition of H3K4me3 and H3K27ac, decreased H3K4me1, and increased transcription of enhancer RNAs (eRNAs) and nearby genes [25]. In DU145 prostate cancer cells, in addition to KDM5C, ZMYND8 interacts with KDM5D, to act as transcriptional co-repressors, involved in regulating metastasis-linked genes (Figure 3C) [8]. ZMYND8 and KDM5D act as general negative regulators of enhancers in prostate cancer cells, and they antagonize the expression of these genes by recognizing the gene-activation-related dual-histone marker H3K4me1-H3K14ac [8].

## 4. Tumor Suppression by ZMYND8

The expression of ZMYND8 decreases in some cancers [10,25,36]. It is downregulated in invasive ductal and lobular breast cancer tissues, compared with normal tissues [10]. In addition, ZMYND8 expression is lower in breast cancer patients with invasive ductal carcinoma than in ductal carcinoma in situ [25]. In 190 patients with nasopharyngeal carcinoma, low ZMYND8 expression was correlated with late T stage, presence of lymph node metastasis, advanced stage, and poor overall patient survival [36]. ZMYND8 is a retinoic acid–inducible gene, and ATRA, a differentiation-inducing drug, can reprogram the epigenetic features of the upstream regulatory region of ZMYND8 and promote its expression [4,9]. On the other hand, ZMYND8 can facilitate the regulation of ATRA-responsive genes in SH-SY5Y neuroblastoma cells [4]. Differentiation of neuronal precursor cells induced by ATRA also requires transcriptional regulation, mediated by the ZMYND8–P-TEFb complex [13]. In MDA-MB-231 breast cancer cells, ZMYND8 upregulates differentiation genes and induces cellular differentiation [11]. The induction of breast cancer cell differentiation by ZMYND8 was noted through the H3K36me2/H4K16ac reader function of ZMYND8 [11]. Microarray analysis of breast cancer cells with ZMYND8 knockout by siRNA revealed that depletion of ZMYND8 reduces the expression of terminal differentiation markers, such as epithelial cell adhesion molecule (EPCAM) and cytokeratin 18 (CK18), by 80% and 86%, respectively [11]. In contrast, overexpression of ZMYND8 induces a 1.5-fold increase in EPCAM and CK18 levels [11]. In addition, ZMYND8 knockdown downregulates the stemness-related genes, prevents tumor cell differentiation, and maintains cancer cells in an undifferentiated state [11]. An in vivo study also showed that ZMYND8 overexpression significantly reduces the subcutaneous 4T1 murine breast cancer growth and increases the expression of differentiation-related genes, including *CK5*, *CK18*, *CK19*, and *EPCAM* in Balb/c mice [11]. All of these results suggest that, in cancer cells, ZMYND8 positively regulates the expression of differentiation-promoting genes and induces differentiation [11].

ZMYND8 also influences cancer cell proliferation. In HeLa and MCF7 breast cancer cells, knocking down ZMYND8 increases the proliferation by about two folds, whereas ZMYND8 overexpression reduces it by about 2.5 to 3 folds [10]. ZMYND8 can be directly recruited to proliferation-promoting genes, including *Ki67* and proliferating cell nuclear antigen (*PCNA*), and affect their expression [10,11]. ZMYND8 knockdown increases the expression of *Ki67* and *PCNA* by about 14 and 4 folds, respectively, in HeLa cells; about 8 and 3 folds in MCF7 breast cancer cells; and about 9 and 2.5 folds in T47D metastatic breast cancer cells [10]. In contrast, overexpression of ZMYND8 reduces the expression of *Ki67* and *PCNA* to 0.1–0.4 fold in these cells [10], and reduces the uptake of bromodeoxyuridine (BrdU) in HeLa cells [10]. However, deletion of ZMYND8 by short hairpin RNA (shRNA) does not affect the proliferation of DU145 prostate cancer cells significantly [8]. Knocking out ZMYND8 can enhance the tumor growth in a mammary fat pad xenograft model of ZR-75-30 breast cancer cells [25]. In contrast, invasive MCF-7 or 4T1 breast cancer cells overexpressing ZMYND8 show a reduction in the tumor size and tumor weight in mice, compared with the control [10,11]. ZMYND8 modulates cell-cycle progression, and its overexpression reduces the *Cyclin* genes, including *G1/S-specific cyclin-E1 (CCNE1)*, *CCNA2*, and *G2/mitotic-specific cyclin-B1 (CCNB1)* in HeLa cells; meanwhile, the inhibition of ZMYND8 by siRNA upregulates their expression [10]. However, in breast cancer cells, the expression of CDKN1A mRNA was upregulated by ZMYND8 loss, which suggests ZMYND8 depletion can increase the p21, which is an inhibitor of cell-cycle progression [20].

In addition to the suppression of cell proliferation, ZMYND8 also affects the tumor cell migration and invasion [8,10]. It can repress the expression of genes that promote metastasis and invasion, and enhance the transcription of epithelial genes [8,10]. In the wound-healing assay, ZMYND8 silencing causes faster wound closure, and its overexpression inhibits this process [10]. In a three-dimension-based assay, ZMYND8-null ZR-75-30 breast cancer cells show increased anchorage-independent growth, migration, and invasion, which can be reversed by restoration of ZMYND8 [25]. In the Matrigel invasion assay, ZMYND8 knockout results in a 1.6-2-fold increase in the invasiveness of HeLa and MCF7 breast cancer cells, and its overexpression reduces the invasiveness by 1.3–1.5 folds in these two cells [10]. Suppression of migration and invasion by ZMYND8 in breast cancer cells is through cooperation with KDM5C and modulation of SA100, as knockout of ZMYND8 or KDM5C can de-repress S100A [25].

The influence of ZMYND8 on cancer cell invasion and metastasis is related to the interaction between ZMYND8, TROJAN, and ZNF592 in breast cancer [24]. Both TROJAN and ZNF592 are the binding partners of ZMYND8, and the function of ZMYND8 is affected by the competitive binding of TROJAN and NF152 with it [24]. TROJAN, an endogenous retrovirus-derived long noncoding RNA, is upregulated in multiple cancer cell lines and is highly expressed in human triple-negative breast cancer [24]. ZNF592 can prevent ZMYND8 degradation, and TROJAN interferes with its ability to bind ZMYND8 [24]. In MDA-MB-231 LM2 breast cancer cells, TROJAN binds to ZMYND8, and increases its degradation through the ubiquitin-proteasome pathway, by repelling ZNF592 [24]. TROJAN knockdown increases ZMYND8 protein, while overexpression of TROJAN or knockdown of ZNF592 decreases it in MDA-MB231 LM2 cells [24]. Both TROJAN and ZMYND8 occupy epidermal growth factor receptor (EGFR), vascular endothelial growth factor-A (VEGF-A), and mouse double minute 2 homolog (MDM2) promoters, suggesting that they co-regulate these metastasis- and angiogenesis-related genes [24]. In an in vivo study, mice intravenously injected with MB-231 LM2 cells with TROJAN knockdown showed fewer metastatic lung nodules, as compared with the control, whereas mice injected with ZMYND8-inactivated cells showed more of them [24]. Increased metastatic ability of MDA-MB-231 LM2 breast cancer cells, induced by ZMYND8 knockdown, can be partially reversed by simultaneous knockdown of TROJAN [24]. In human breast cancer tissues, the expression of TROJAN negatively correlates with ZMYND8 expression, and it positively correlates with EGFR, VEGF-A, and MDM2 expression [24]. In addition, the TROJAN–ZMYND8 combined expression signature can be used to predict the relapse-free survival and overall survival of patients with triple-negative breast cancers (TNBCs); those with high TROJAN and low ZMYND8 expression have worse prognosis, and the ones with low TROJAN and high ZMYND8 levels survive better [24].

ZMYND8 also prevents metastasis of prostate cancer cells [8]. Knockdown of ZMYND8 increases the invasiveness of DU145 and CWR22Rv1 human prostate cancer cells *in vitro*, and this was also noted in an intravenous mouse xenograft model [8]. In mice injected with luciferase-expressing DU145 prostate cancer cells with ZMYND8 silencing by shRNA (shZMYND8), the luciferase signals were about five-fold higher than in those treated with the control shRNA, eight weeks after injection [8]. Histological examination of the lungs confirmed tumor development in the shZMYND8 group [8]. These results suggest that ZMYND8 suppresses invasiveness and metastasis of prostate cancer cells, both in vitro and in vivo [8]. The ZMYND8-induced suppression of invasiveness and metastasis in prostate cancer cells was demonstrated to be through cooperating with its transcriptional corepressor KDM5D [8]. They co-occupy multiple metastasis-linked genes, such as *SAIL2, CD44, VEGF*, and *EGFR*, and hinder the expression of these genes by interacting with the gene-activation-related dual-histone mark H3K4me1-H3K14ac [8]. Knockdown of either ZMYND8 or KDM5D can upregulate these metastasis-related genes [8]. KDM5D levels are significantly reduced in metastatic prostate tumors (100%, 6/6) and primary prostate tumors (41%, 28/68), as compared with normal tissues (10%, 2/21) [37]. These observations suggest that cooperation between ZMYND8-mediated recognition of H3K4me1-H3K14ac and KDM5D-catalyzed H3K4 demethylation can hamper the expression of metastasis-linked genes [8,37]. H3K4me1-H3K14ac may act as a poised epigenetic signature, and it is converted to active dual marker H3K4me3-H3K14ac during cancer metastasis [8]. Therefore, the epigenetic switch is critical for regulating the expression of metastasis-linked factors, including Slug, CD44, VEGFA, and EGFR, which, in turn, modulate the invasiveness of prostate cancer cells [8].

ZMYND8 can regulate the epithelial–mesenchymal transition (EMT), which is important for cellular invasion [10]. It regulates EMT genes through recognizing H3K36me2/H4K16ac on respective genes [10]. The epithelial phenotype of cancer cells is governed by claudin-1 *(CLDN1),* claudin-7 *(CLDN7),* and E-cadherin *(CDH1)* genes, while the mesenchymal phenotype is regulated by *ZEB1*, *SNAI1*, *SNAI2*, and Vimentin *(VIM*) genes [10,38]. In MDA-MB-231 breast cancer cells, ZMYND8 is directly recruited to metastasis-linked genes *SNAI2* and *TWIST1*, to regulate EMT [11]. Overexpression of ZMYND8 reduces the expression of *VIM*, *SNAI1*, and *ZEB1* and increases that of *CLDN1* and *CDH1* in MCF7, HeLa, and T47D cells [10]. ZMYND8 knockdown reduces the expression of *CLDN1/7* and *CDH1* genes in MCF7 and HeLa cells, and that of *CDH1* and *CLDN7* in T47D cells, while it increases the expression of *VIM*, *SNAI1*, and *ZEB1* in MCF7, HeLa, and T47D cells [10]. These observations suggest that ZMYND8 is involved in maintaining the epithelial cell phenotype, and the deletion of ZMYND8 enhances the mesenchymal transition [10].

Overall, ZMYND8 appears to act as a tumor suppressor in breast, prostate, and nasopharyngeal cancers [8,9,24,25]. It can induce differentiation; inhibit cell proliferation, cell-cycle progression, invasiveness, and metastasis; and maintain the epithelial phenotype in these cells [8,9,24,25].

## 5. Pro-Oncogenic Effects of ZMYND8

The level of ZMYND8 expression is crucial for proliferation and invasiveness of cancer cells, and increased ZMYND8 expression is reported in colorectal, cervical, breast, and prostate cancers [2,21,23,36]. Analyses of the Cancer Genome Atlas (TCGA) and Gene Expression Omnibus (GEO) database suggested that high expression of ZMYND8 was closely correlated with poor overall and disease-free survival in 174 colorectal cancer patients [23]. Increased ZMYND8 expression was linked to the high-grade cervical intraepithelial neoplasia and cervical carcinoma [21], zebrafish prostate cancer DU145 xenografts, and prostate cancer tissues from patients [2]. In addition, ZMYND8 expression is inversely correlated with metastasis-free survival in breast cancer patients, with high ZMYND8 expression being associated with shorter survival of patients with breast cancer [22,27]. However, increased ZMYND8 expression is associated with high mortality in patients with different subtypes and stages of breast cancer, but not with different grades of tumor [22]. ZMYND8 is a downstream target of estrogen receptor α (ERα), and it is involved in a positive feedback circuit of the ER pathway [27]. It is highly expressed in ER-positive and triple-negative breast cancers [22,27]. Amplification and overexpression of ZMYND8 are more frequent in luminal B breast cancer subtypes [22,27]. Tissue microarray analysis of 160 human TNBC specimens and 91 paired adjacent normal breast tissues revealed that 80% of tumors showed an increased expression of ZMYND8, with 50% showing moderate expression and 30% showing high expression [22]. In contrast, only 18% of the normal tissues had moderate ZMYND8 expression and none showed high expression [22]. An in vitro study also showed that ZMYND8 knockdown by small interfering RNA (siRNA) suppressed colony formation and reduced the number of MCF-7 breast cancer cell colonies; however, ZMYND8 knockdown did not affect the proliferation rate of the breast cancer cells [22]. In vivo experiments revealed that ZMYND8 increased the circulating breast cancer cells, to promote their extravasation and colonization, leading to lung metastasis in severe combined immunodeficiency (SCID) mice, whereas in ZMYND8 knockouts, these features were reversed [22].

**Figure 4:**
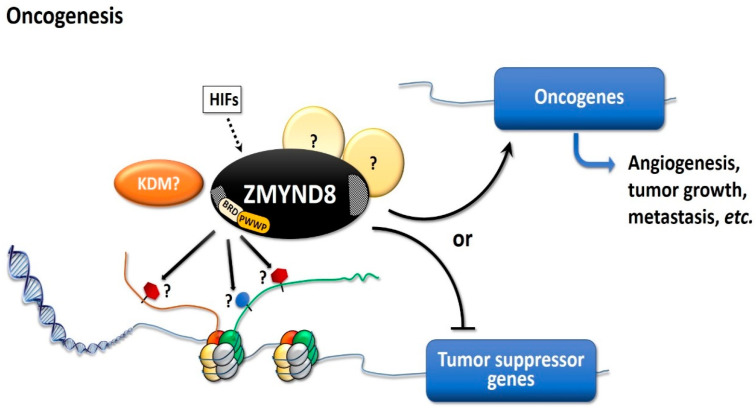
Diagram showing the possible mechanisms of the pro-oncogenic effects of ZMYND8. ZMYND8 can be activated by HIFs and induces angiogenesis, cellular proliferation, and tumor growth. The pro-oncogenic pathways of ZMYND8 are still unclarified. ZMYND8 may cooperate with demethylase, recognize the histone marks, suppress the transcription of tumor suppressor genes, or activate oncogenes, to enhance the tumorigenesis. HIFs: Hypoxia-inducible factors; KDM: Lysine demethylase.

ZMYND8 may induce angiogenesis in cancer, especially in hypoxic conditions (Figure 4). Under hypoxia, ZMYND8 expression was induced, together with hypoxia-inducible factor-1α (HIF1α) and VEGF, at 72 h post-fertilization in zebrafish [2]. In breast cancer cells, HIF-1 and HIF-2 can induce ZMYND8 expression, and HIFs and ZMYND8 co-activate oncogenes and increase RNA polymerase II phosphorylation, leading to the promotion of cell motility, tumor growth, and lung metastasis [22]. When exposed to hypoxic condition, with 1% O_2_ for 12 days, the MDA-MB-231 breast cancer cells with ZMYND8-knockout showed decreased colony formation, migration, and invasion, compared to parental cells [22]. Furthermore, ZMYND8 can induce the expression of VEGFα mRNA and promote angiogenesis in prostate cancer xenografts in zebrafish and tube formation in human umbilical vascular endothelial cell cultures [2]. In zebrafish, ZMYND8 knockdown suppresses tumor angiogenesis in DU145 prostate cancer xenografts, and the re-introduction of ZMYND8 mRNA restores the tumor angiogenesis [2]. However, ZMYND8 overexpression does not increase VEGFα expression in DU145 xenografts, suggesting that ZMYND8 mainly acts on the surrounding zebrafish tissues, to regulate the expression of VEGFα, to induce angiogenesis [2]. ZMYND8 knockout also decreases the microvessel density in the mammary fat pad MDA-MB-231 tumor and subcutaneous MAF-7 tumor in SCID mice, which further supports the induction of angiogenesis by ZMYND8 [22]. In addition, transcriptome analyses reveal an increase in ZMYND8 expression during tumor angiogenesis in prostate cancer xenografts [2]. Furthermore, ZMYND8 is a binding partner of PKC, and PKC isozymes—in particular, PKCβ—are important mediators of VEGF signaling [2,3,39]. Inhibition of PKCβ causes decreased proliferation of endothelial cells and neovascularization of hepatocellular carcinoma, in a syngeneic xenograft model in BALB/c mice [39]. Thus, ZMYND8 can be induced in hypoxic conditions, and it can promote tumor angiogenesis, probably through HIF and VEGF activation, and enhance tumor growth [2,22].

Overall, these results indicate that ZMYND8 may have a crucial role in the tumorigenesis of breast, prostate, colorectal, and cervical cancers [2,21,22,23]. In addition, ZMYND8 may promote angiogenesis in zebrafish, as well as breast and prostate cancers [2,22]. Therefore, ZMYND8 is important for tumor cell proliferation, angiogenesis, and tumor growth [23]. However, data supporting its oncogenic role are still limited and the pro-oncogenic pathways of ZMYND8 are still unclarified (Figure 4).

## 6. Conclusions

**Figure 5:**
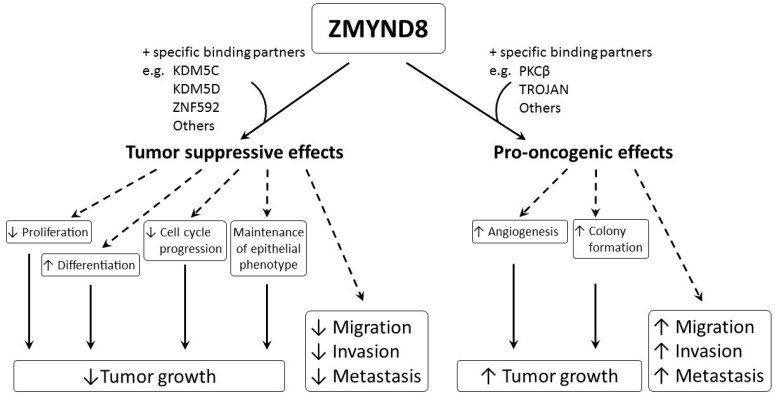
Diagram showing the pro-oncogenic and tumor-suppressive effects of ZMYND8 in cancers. The functions of ZMYND8 were considered through the interaction between ZMYND8 and a specific ZMYND8-binding partner. The ↑ indicates enhancement, and ↓ indicates suppression.

Aberrant regulation of gene expression and epigenetic mutations play an important role in carcinogenesis. ZMYND8 is a transcription factor, a histone H3-interacting protein, and a putative chromatin reader/effector, important for regulating transcription in normal cells, and its mutation, altered expression, and fusion with other genes, are associated with development and progression of cancer. Increased expression of ZMYND8 is associated with breast, prostate, colorectal, and cervical cancers, and it is pro-oncogenic in breast and prostate cancers [2,21,22,23]. ZMYND8 is induced during hypoxic conditions, and can promote angiogenesis in zebrafish, as well as in breast and prostate cancers [2,22]. In contrast, lower expression of ZMYND8 is linked to breast, prostate, and nasopharyngeal cancers, and ZMYND8 exerts tumor-suppressive effects on breast and prostate cancers [8,9,24,25]. It suppresses tumor growth by promoting differentiation; inhibiting proliferation, progression of cell cycle, invasiveness, and metastasis; and maintaining the epithelial phenotype in various types of cancer [8,9,24,25]. Although all of these results indicate that ZMYND8 is important in cancer, they are contradictory, and the actual role of ZMYND8 in cancer is still unclear. In addition, the investigations are limited to a few types of cancer, like that of breast and prostate. As ZMYND8 interacts with various transcriptional corepressors, chromatin remodeling complexes, histone demethylase/deacetylase, and acetyl transferase enzymes, the role of ZMYND8 in cancers may be related to not just its direct action, but to multiple factors. Different cell types, fusion with other genes, and the interaction of ZMYND8 with various binding partners may affect the functions of ZMYND8 in cancers. The interaction between ZMYND8 and various binding partners is considered to be crucial in determining whether the function of ZMYND8 is pro-oncogenic or tumor suppressive (Figure 5).

For example, the interaction between ZMYND8 and PKCβ or TROJAN may induce pro-oncogenic effects, while interaction between ZMYND8 and KDM5C, KDM5D, or ZNF592 may cause tumor-suppressive effects. Therefore, more studies are necessary, and they need to be focused not only on ZMYND8, but also on its specific binding partners in different cancer types. Hopefully, in the future, regulation of the expression of ZMYND8 and/or its binding partners may become useful in treating cancer.

## Sample Availability

Samples are not available.
